# International trends in the implementation of assessment for learning *revisited*: Implications for policy and practice in a post-COVID world

**DOI:** 10.1177/14782103241255855

**Published:** 2024-05-22

**Authors:** Louis Volante, Christopher DeLuca, Nicole Barnes, Menucha Birenbaum, Megan Kimber, Martha Koch, Anne Looney, Jenny Poskitt, Kari Smith, Claire Wyatt-Smith

**Affiliations:** Faculty of Education, 7497 Brock University, St. Catharines, ON, Canada; 4257 Queen’s University, Kingston, ON, Canada; 8087 Montclair State University, Montclair, NJ, USA; School of Education, 26745 Tel Aviv University, Tel Aviv, Israel; 1969 Queensland University of Technology, Brisbane, QLD, Australia; 8664 University of Manitoba, Winnipeg, MB, Canada; 8818 Dublin City University, Dublin, Ireland; 6420 Massey University, Palmerston North, New Zealand; 8018 Norwegian University of Science and Technology, Isdalstø, Norway; 95359 Australian Catholic University, Brisbane, QLD, Australia

**Keywords:** Assessment for learning, education policy, education reform, artificial intelligence

## Abstract

This paper discusses the evolution of assessment for learning (AfL) across the globe with particular attention given to Western educational jurisdictions. Scholars from Australia, Canada, Ireland, Israel, New Zealand, Norway, and the United States discuss prominent assessment reforms within their respective countries over the last decade. Particular attention is given to the impact of the pandemic as well as technological developments for classroom assessment policies and practices. Ongoing tensions that exist between AfL and summative forms of assessment within national policy initiatives are also revisited in relation to the seminal version of this article published 10 years ago.

## Introduction

Although operational definitions vary amongst different researchers, there is general consensus that Assessment *for* Learning (AfL) includes assessment practices that are ongoing and take place during a lesson or unit of study (DeLuca and Volante, 2016). AfL practices often include student self-assessment, peer-assessment, goal-setting practices, and methods to track students’ understanding such as classroom observations, questioning techniques, and/or the sharing of success criteria (Klinger et al., 2012). When used consistently, AfL practices have shown promise in raising student achievement and learning outcomes around the world (see De Vries et al., 2022; William et al., 2010).

The research literature is equally clear that implementing AfL is not without its challenges. In particular, policy contexts that emphasize summative forms of assessment, for example, high-stakes testing contexts (OECD, 2023), can undermine AfL efforts. Coupled with recent technological advances such as artificial intelligence (AI) applications, which jeopardize academic integrity (Volante et al., 2023a), along with the deleterious impact of the pandemic on student learning, particularly students from lower socioeconomic backgrounds (Schnepf et al., in press), there is a pressing need to more carefully support AfL across education systems, both in policy and practice.

This paper considers the nexus of the aforementioned teaching and learning challenges and discusses recent AfL reforms in a cross-section of seven countries: Australia, Canada, Ireland, Israel, New Zealand, Norway, and the USA. Scholars from these nations revisit some of the key international trends articulated in our previous publication “International trends in the implementation of assessment for learning: Implications for policy and practice” published a decade ago in *Policy Futures in Education* (see Birenbaum et al., 2015). Each of the ensuing national profiles provides a brief synopsis of the evolution of AfL reforms since that time and discusses the relative effectiveness of policy developments to support student achievement and learning in a post-COVID world. In doing so, this article offers policymakers a critical analysis of salient policies and practices related to AfL across education systems internationally.

## International profiles

### United States

Formative assessment is used widely in the United States (US), which is evidenced by its inclusion in both policy and practice. For example, the Council of Chief State School Officers and the Formative Assessment for Teachers and Students State Collaborative on Assessment and Student Standards have both commissioned guidelines and resources for observations and reflections on formative assessment practices (Yan et al., 2021). These guidelines address various aspects of formative assessment, including the personal and contextual factors that influence its implementation. Moreover, formative assessments are used to target instructional practices to meet specific student needs and monitor student progress toward valued state learning outcomes (Yan et al., 2021).

In practice, formative assessment takes various forms such as checklists, rubrics, written papers or oral presentations, graphic organizers, Socratic questioning, and more (Yan et al., 2021). These practices are often embedded in teacher observations of student performance, teacher questioning/class discussions, analysis of student work, student self-assessment, and student journals (Yan et al., 2021). The concept of formative assessment is also reflected in the feedback process for multiple paper drafts, criterion discussions during 1-on-1 conferences, and regular online quizzes (Yan et al., 2021).

However, despite the well-documented benefits of formative assessment on teaching and learning (Black and Wiliam, 1998; William, 2010), its adoption in classrooms remains less than satisfactory (Schotter et al., 2010; Yan and Brown, 2021). This is due to a variety of personal and contextual factors (Yan et al., 2021). A systematic review of 52 studies identified major personal factors influencing teachers’ intentions to conduct formative assessment, including instrumental attitude, self-efficacy, and education and training. Contextual factors such as internal school support, external policy, school environment, and cultural norms were also reported (Yan et al., 2021). Despite these challenges, the benefits of formative assessment, which vary widely among different implementations and student populations (Bennett, 2011), have made it an important agenda in educational reform in the US (Birenbaum et al., 2015).

That said, the COVID-19 pandemic has fundamentally reshaped the landscape of education in the US, particularly in the realm of assessment. This shift, which emphasizes understanding students’ needs and improving learning rather than merely grading work, requires collaboration among teachers, administrators, parents, and students (Goble, 2022). Technological developments, such as AI, have facilitated the rise of digital formative assessment (DFA), which has seen increased use since the start of the pandemic (Çekiç and Bakla, 2021; See et al., 2022). DFA tools, including popular open-access platforms like Kahoot, Mentimeter, and Edulastic, allow for the collection of real-time student responses, enabling timely feedback and individualized instruction based on students’ immediate needs (Çekiç and Bakla, 2021). However, the effectiveness of these tools is still under investigation. A systematic review by See et al. (2022) found support for the use of DFA in improving learning outcomes in mathematics and reading for early grades, but not for writing, and the evidence was inconclusive for science. The authors called for more rigorous, large-scale studies before recommending widespread use of DFA in schools (See et al., 2022). The future of learning and assessment in the U.S. will likely depend on the US’s continued recovery from the pandemic, which is still yet to be seen (Jiao and Lissitz, 2020).

With regard to the relationship between formative and summative assessment in the US, formative and summative assessments are not mutually exclusive. Rather they exist on a continuum, with the nature of the assessment determined by the context’s needs and practical implications (Dolin et al., 2018). This spectrum reflects the inherent connection between these two types of assessments, a connection that must be grounded in the factors of the learning and teaching context (Lau, 2016). This connection can be conceptualized in two ways: the linkage between formative and summative assessments, and the integration of both types within a single assessment. The former refers to the alignment of the assessment’s purpose with the validity derived from its summative components and the reliability gleaned from its formative elements. The latter involves utilizing evidence from a single assessment to serve both formative and summative purposes. However, achieving this integration is challenging. Success requires clear goal setting that aligns with inquiry-based learning activities, employing a variety of methods, and involving students in assessing their own learning progress (Dolin et al., 2018). In the context of large-scale testing programs, the synergy between formative and summative assessments becomes particularly critical. The balance between these assessments can significantly impact the effectiveness of the educational process, shaping not only how learning is measured but also how it is facilitated and enhanced.

### Canada

In Canada, each province and territory develop their own K-12 curriculum standards and assessment policies for implementation. These documents are revised over time, often after the political party which forms the government changes. For example, at this time, Alberta is moving toward increased emphasis on standardized testing alongside a back-to-basics approach to curriculum (Volante and Mattei, 2024) while changes to British Columbia’s assessment policy reflect the competency-based curriculum they recently implemented (Murai and San Juan, 2024). Within this decentralized system, most policies state that the main purpose of assessment is to support student learning. Some policies require the use of AfL and include steps for educators to follow (e.g., Ontario Ministry of Education, 2010). Others require practices such as providing individualized feedback and developing students’ self-assessment skills without mentioning AfL (e.g., Province of Nova Scotia, 2021). Most policies identify fairness as a guiding principle even though enhancing fairness in AfL and summative assessment remains a concern (Rasooli et al., 2023). Thus, AfL is evident in policies and guidelines across Canada, albeit expressed in varied ways. Notably, while requiring AfL practices, these documents continue to focus overwhelmingly on grading, report cards, and provincial assessments. This creates a sense of inconsistency that adds to the tensions educators experience as they use AfL in their classrooms (Suurtamm and Koch, 2014).

In the midst of these challenges, AfL policy and practice has continued to develop in encouraging ways. For instance, many provinces now call for assessment to reflect culturally responsive and sustaining pedagogies. This approach, sometimes called culturally responsive assessment (CRA), advances many AfL principles while foregrounding the need to respect the perspectives and experiences of First Nations, Inuit, and Métis peoples. CRA also helps educators consider the diverse cultural and linguistic backgrounds of students in Canadian classrooms. Recent examples of CRA include guidelines for enacting culturally inclusive assessment released by several provinces; resources developed by University of British Columbia researchers to help mathematics educators enact CRA (https://elvlc.educ.ubc.ca/culturally-responsive-mathematics-assessment/); and articles describing researcher-practitioner initiatives in various provinces that center on Indigenous approaches to teaching, learning, and assessment. In addition, some teacher education programs are working to “decolonize assessment” through increased use of formative assessment (Hill et al., 2024: p.112).

Other studies have demonstrated innovative ways that AfL is being enacted in various Canadian school districts. For instance, Grades 4 to 12 teachers in two Ontario districts increased formative assessment in their mathematics classrooms despite dilemmas that arose as they made these changes (Suurtamm and Koch, 2014). In Ontario and British Columbia, teachers leveraged the assessment opportunities of “maker-centered learning” to gain a deeper sense of student learning and foster self-assessment (Murai and San Juan, 2024). In Quebec, a survey of 223 teachers about their assessment practices in health education found that 88.6% incorporated self-assessment while 47.5% included peer-assessment (Bezeau et al., 2023). In Manitoba, increased use of AfL accompanied by a reduced emphasis on grading is being adopted in secondary schools in some districts (Koch & Potapinski, forthcoming) while teachers and researchers in other parts of Canada are developing resources for the use of artificial intelligence in AfL (Volante et al., 2023b). Each of these studies also highlights the need for teachers to have ongoing support as they enact AfL. Most schools, districts, and teacher associations recognize this need and regularly offer learning opportunities focused on aspects of AfL. In addition, a non-profit organization aptly titled the *Canadian Assessment for Learning Network* (https://www.cafln.ca) was established in 2013 to support educators who want to more fully implement AfL in their setting.

Thus, AfL policy and practice in Canada is a work-in-progress fueled by the efforts of teachers, school and district leaders, professional learning providers, and researchers. Nevertheless, tensions between AfL, grading, and large-scale assessment (LSA) remain. During the pandemic, provincial assessments were discontinued in most jurisdictions and the validity of these assessments and other summative assessment practices were briefly reconsidered, particularly for disadvantaged students. Most jurisdictions have now reinstated national and provincial assessments despite research showing concerns with their accuracy (Miller and Yan, 2023). At the same time, there are signs that the dichotomy between AfL and summative forms of assessment is diminishing. For instance, Manitoba’s provincial assessment program includes formative assessments in mathematics, reading and writing which take place at the start of Grades 3 and 7. Learning objectives to be assessed and guidelines for evaluating student achievement are provided to inform instructional planning but standardized test items are not administered. Another promising development is that assessment policies in Canada increasingly describe grading as a process where teachers use their professional judgment to consider many forms of evidence including classroom observations and student’s perspectives on their learning. These changes suggest an increased appreciation of how AfL can contribute to learning and a much more nuanced understanding of how AfL relates to other forms of assessment.

### Australia

In Australia, AfL has become less prominent in education policy and curriculum documents in recent years. When AfL is used, it tends to refer to “teachers using evidence about students’ knowledge, understanding and skills to inform their teaching” (New South Wales Education Standards Authority, 2022, paragraph 4). Teachers are advised that AfL “usually occurs throughout the teaching and learning process to clarify student learning and understanding” (paragraph 4). AfL has been associated with formative assessment. For example, the NSW Education Authority (2022) states that AfL is “sometimes referred to as formative assessment” and includes “clear goals for the learning activity; provides effective feedback that motivates the learner and can lead to improvement; reflects a belief that all students can improve; and encourages self-assessment and peer-assessment as part of the regular classroom routines” (NSW Education Standards Authority, 2022, paragraphs 2-3).

There has been increasing emphasis on teacher, self, and peer feedback in helping students to improve learning. This focus is clear nationally, as illustrated by Education Services Australia [ESA] (n.d), and across many states including Queensland and Western Australia. In WA, the School Curriculum and Standards Authority (2024) indicates “assessment must be educative,” promoting learning through teacher feedback that aids students and helps teachers to plan further learning.

Interestingly, the Australian Professional Standards for Teachers (AITSL, 2018) emphasize curriculum standards and feedback in teacher practice, albeit there is no reference to AfL. It is referenced, however, on ESA’s (n.d.) website. The “Planning for Assessment” resource includes questions for teachers probing if they have identified formative or summative purposes for assessment, and identified where they can collect evidence of *assessment of learning*, *assessment for learning*, and *assessment as learning*.^1^ Recent nationally funded research with teachers also showed the benefits of personalized feedback provided to students verbally, in writing, or through learning analytics (Adie et al., 2023).

#### Recent innovations in AfL

Notable developments have occurred in formative assessment and AfL driven by government and commercial entities. Two examples are illustrative: the *National Formative Assessment Resource Bank*; and *Essential Assessment*. The Bank’s aims include a digital portal to collect and collate evidence about students’ learning and plotting against learning progressions. Trials in Australian schools are underway in English and Mathematics and include Foundation to Year 10 (ACARA, 2024).

*Essential Assessment* (2024) offers online formative and summative literacy and numeracy assessments aligned to version 9 of the Australian Curriculum, the New South Wales syllabus, and the Victorian curriculum. Using this tool, teachers can undertake a range of formative assessment actions including pre-assessing each student online, using content descriptions and proficiency strands, and obtaining an achievement level or a common grade.

*Synergy between AfL and summative forms of assessment.* This account reveals how the affordances of technology are enabling new synergies across AfL, formative, and summative assessment. The National Assessment Program—Literacy and Numeracy (NAPLAN) is a further illustrative case, with census testing in years 3, 5, 7, and 9.

Recently, Computer Adaptive Testing (CAT) including branching was used in the design of NAPLAN Online. Branching occurs when an algorithm is used to adjust test items to the test taker (student). The adjustments depend on the student’s success in responding to earlier and later test questions. CAT is intended to improve students’ experience of test taking. Further, the move to NAPLAN fully online has enabled results for reading, numeracy, spelling, grammar, and punctuation to be returned to schools earlier in the school year than was previously possible.^2^ Branching and the timely return of results suggest a policy turn to NAPLAN being used for formative purposes (McGraw et al., 2020).

The shifting status of AfL and formative assessment in an Australian education landscape has been set against a backdrop of changing digital technologies. Recent research has mapped the evolution of digitally mediated ways to assess student progress authentically in context (Wyatt-Smith et al., 2019, 2021). As we move from Computer-Based Testing (CBT) to Computer Adaptive Testing (CAT), and on to Immersive Assessment (IA) using Artificial Intelligence and 3D, further changes in the meaning and practices of AfL are anticipated.

### New Zealand

Assessment for learning persists in Ministry of Education assessment policy, resources, and school practice (New Zealand Ministry of Education, 2019), though less consistently than a decade ago. Attention was diverted to National Standards until 2018, along with strategies to address student achievement inequities, cultural responsiveness, managing schooling and student wellbeing during COVID-19 disruptions, adapting to online pedagogy and assessment, and responsiveness to emerging technologies (Bonne and Wylie, 2017; Hipkins and Cameron, 2018). While some schools managed to reconcile accountability demands with assessment for learning, teacher attention was largely focused on teaching and assessing “priority learners” who were performing “below” or “well below” the National Standards, which 69% of teachers reported narrowing their teaching of the curriculum (Bonnie and Wylie, 2017). Only “34% of [primary] principals reported a school-wide focus on AfL in 2013, and in 2019, 39% of principals” (Wylie and MacDonald, 2020: p. 66).

Data from National Standards, NZ’s National Monitoring Study of Student Achievement, PISA, and TIMSS (Poskitt, 2023) fueled concerns about persistent inequities for groups of students, including Māori, Pacific, low SES, and students with complex learning needs. These concerns led to policy and professional learning priorities of literacy and numeracy, and culturally responsive teaching to lift achievement. The effect on AfL was variable. Hipkins and Cameron (2018) argued that diversions of teacher time led to “inadequacies in New Zealand teachers’ assessment knowledge and skills and weakened ability to use both formal and informal classroom assessment data” (p.11). Yet 60% of teacher respondents in the 2019 National Survey of NZ primary teachers claimed their students “were taught strategies to assess and modify their own learning and to assess their peer’s work,” and “around half the teachers reported that most or all of their students were regularly involved in monitoring and assessing their own progress” (Wylie and MacDonald, 2020: p.57). Nevertheless, at only 15% for lower decile 1–6 schools, patterns of use varied significantly.

#### Innovations

Since 2019, societal changes have added further challenges for AfL: advancements in technology, inclusive accommodations of diverse learners such as [dis]abilities, fluid gender and cultural identities, greater sensitivity to student wellbeing and voice, and the COVID pandemic (Poskitt, 2023). The New Zealand Council for Educational Research (NZCER), who develop many of the assessment tools used in schools, incorporated principles of equity, social, and cultural diversity in tool content, design, and layout. Computer adapted content improved accessibility for students with visual, auditory, and sensory requirements, while online assessments increased school access and adaptability to resources like online marking services and guidelines for teachers using assessment data to inform learning and teaching (NZCER, 2023).

Similarly, the New Zealand Qualifications Authority enabled students to submit electronic internal assessment evidence such as portfolios and created online digital examinations for the National Certificate of Educational Achievement (NCEA). Digital examinations are more aligned to contemporary ways of learning and teaching, can be adapted for diverse student needs, increase system resilience in pandemic times (Poskitt, 2022b), and provide data to schools for interpretation and improvement.

However, technological innovations have limited value unless educators have appropriate knowledge, skill, and resources to interpret and use the resulting information. McKenzie (2023) investigated secondary school use of NCEA summative data to inform learning and teaching programmes. He described various complexities with effective use of assessment information—including ill-defined data literacy competencies, blaming of limited time and resources for minimal engagement with data, sensitivities around engaging staff in thorough examination of data and teaching practices, and scarcity of strategic planning for prioritizing attention to evidence-informed improvements.

#### Where next for AfL in New Zealand?

Although pockets of effective AfL practice are occurring in primary and secondary schools, and across Kāhui Ako/communities of schools (Hipkins and Cameron, 2018), to scale up such practices, assessment capabilities must be explicitly fostered during Initial Teacher Education (Dixon and Hawe, 2018), extended through ongoing professional learning (Poskitt, 2023) and supportive school systems (Hipkins and Cameron, 2018).

School systems can be supported through empowering school curriculum (Chamberlain et al., 2021). Considerable time and effort have been devoted to refreshing the New Zealand Curriculum, *Te Mātaiaho* (New Zealand Ministry of Education, 2023), which is strengths-based, aiming to address equity and wellbeing, inclusivity for every learner, valuing students’ cultural identity and language, and a broader understanding of success. A refreshed Assessment Position paper was drafted in 2023, though it and further development of the new curriculum have been delayed by governmental changes and fiscal restraint.

One counter measure to political forces was the development of an assessment network, the New Zealand Assessment Institute (NZAI), established in 2018. Members of the network include “academics, professional development facilitators, teachers, and principals, as well as personnel from government agencies including the Education Review Office, MOE, NZCER and NZQA” (Poskitt, 2018: p. 106). Through regular webinars, annual conferences (in which keynote speakers stimulate thinking and workshop presenters share effective practices), access to resources and research on their website, as well as member involvement in national and international Advisory Groups to government bodies, there is potential to effect change at the policy maker, policy influencer (researchers, professional development, Initial Teacher Education, educational agency), and policy enactor (school and community) levels (Poskitt, 2018). In an era of AI, geo-political instability, serious climatic and economic threats, the decade ahead will present ongoing challenges to AfL, so only with the test of time will the effectiveness of initiatives, like the system-wide collaboration of NZAI, be known.

### Republic of Ireland

Determining the status of AfL in the Irish context is not straightforward. In the classrooms of lower secondary education, practices associated with AfL appear to be embedded. The new framework for primary education (Irish Department of Education, 2024) makes no explicit reference to AfL, but stresses the important role of pupils as collaborators in assessment processes, and the place of assessment as integral to teaching and learning. However, beyond advocating for greater attention to formative assessment, the latest publication on senior cycle reform (NCCA, 2022) reflects the continuing dominant force of the high-stakes examination on that phase of education.

A new framework for the primary school curriculum was published early in 2024. As in the earlier 1999 primary curriculum, assessment is presented as central to teaching and learning. The key principles of the curriculum present assessment as collaborative, integral to high quality teaching and learning, involving “children, teachers, parents and others,” (p.6). Later in the document, assessment is described as a “shared endeavour” (p.21) in which teachers and children work together to use information to inform teaching and learning. The term “feedback” seems to have been displaced by “information,” perhaps to include the full range of stakeholders in the assessment process. The foregrounding of pupils in assessment is noteworthy. It reflects both the underlying emphasis across the new curriculum on the agency and participation of children, and the broader policy emphasis in Ireland on child agency, as reflected in the Looking at our Schools framework (Irish Department of Education, 2022) used in processes of school inspection and evaluation.

Research into the impact of a decade of curriculum and assessment reform in lower secondary education in Ireland is showing that AfL practices, and feedback in particular, are beginning to feature as routine classroom practice. The National Council for Curriculum and Assessment (NCCA), which developed the reforms, publishes what it calls Early Enactment Reports as the changes are introduced across different subjects. The reports on Visual Art and Modern Foreign Languages (2023) offer insights into the positive impact on classroom practice and on student experience of the greater focus on formative assessment, feedback to learners, and sharing criteria for success at the heart of the reforms. The responses of students who remarked on the welcome change from receiving feedback mainly through summative tests, marks, and grades (p. 6) are particularly encouraging for policymakers and advocates for AfL. The report on modern foreign languages notes that the students involved in the review demonstrated familiarity with the language of formative assessment and the concept for features of quality work. Interim results from a more extensive longitudinal study on the introduction of the Framework for Junior Cycle have also reported positive developments in formative assessment. While the interim report (McGarr et al., 2023) indicated that the examination at the end of junior cycle continued to backwash into teaching and learning (p. 17), teachers reported providing greater feedback, particularly more formative feedback, than in the pre-reform era (p, 146).

The reform of upper secondary education in Ireland has had a long and contested history. Its most recent chapter has been the publication of the NCCA advisory report on senior cycle which arose out of extensive consultation and research regarding the future development of that phase of education (NCCA, 2022). The defining feature of that phase of Irish education is the high-stakes examination, the Leaving Certificate. For most students the results determine access to their chosen course in higher education. Similar to the new primary curriculum, the advice on senior cycle describes assessment as integral to teaching and learning, but adds a third process supported by assessment—reporting (p. 29). Perhaps reflective of both the stage of learning and the selection function of the examination results, the section on assessment is entitled *Assessment, Reporting and Transitions* (p. 46). The consultations about assessment in senior cycle highlight the negative backwash of the summative examination and the levels of student stress associated with the test. It is suggested that consideration be given to renewing the focus on formative assessment. There is no reference to feedback, nor to AfL in the document. The response of the Minister for Education to the advice included plans to ensure that the final examinations in any subject would contribute only 60% of the total marks, and to introduce teacher-based components to that examination. Those proposals provoked strong opposition from the teacher unions, but the Minister continued to advocate for her plans until the following September when she announced that the plans had been shelved due to the “accelerated evolution and growth of generative AI.” She confirmed that for the time being at least, all assessments for the Leaving Certificate would be undertaken by the State Examinations Commission. The teacher unions welcomed the announcement. As noted in an Irish daily newspaper, AI fears prompt Minister to suspend plans for teacher-based assessments in Leaving Cert, 2023 (Irish Examiner, 2023).

### Norway

Assessment is frequently discussed at all levels of education in Norway, especially how to link assessment to the curriculum. When the new curricula for all subjects were introduced in 2020, previous forms for assessment had to be revisited. There is a strong emphasis on “in-depth learning” defined as “the gradual development of knowledge and lasting understanding of concepts, methods, and relationships within subjects and between subject areas. This means that to reflect on own learning and use what is learned in different ways in familiar and unfamiliar situations, alone or together with others” (Norwegian Directorate for Education and Training, n.d). In-depth learning is to take place in all subjects, yet it is still unclear how to do it, and how to assess it, specifically how to involve students in their own learning and assessment. Moreover, the new Educational Act states that “students have the right to participate in all that concerns them according to this law, and they have the right to freely state their opinions. The students shall be heard and their opinion will be taken into consideration according to their age and maturity” (Lovdata, n.d).

For more than a decade, all students have the right to formative assessment by law in Norway. There are no grades in the first 7 years of school, and teacher assessment is the main assessment throughout the school system. In lower secondary school (8–10), teacher assessment is translated into a grade every half year. The guidelines say that the teachers’ grade should be based on a combination of assessment approaches to document students’ competencies in various ways, such as observations, dialogues, discussions, performances, oral and written work, and learning logs. The grade must be followed by verbal feedback to the student and the parents as an explanation of the grade (Norwegian Ministry of Knowledge, 2023).

In 2010, a large AfL professional development program was initiated in Norway, under the responsibility of the Norwegian Directorate of Education and Training. Hopfenbeck et al. (2015) carried out a large interview study with various stakeholders of education after 4 years. The main findings were that trust, dialogue and high levels of teacher agency were needed for successful implementation. However, there is a tension between trust and accountability in the system (Hopfenbeck et al., 2015). In a more recent study, Sandvik (2019) studied what AfL communities developed in schools as a result of the large professional development initiative. Sandvik (2019) defined “AfL communities in schools as learning communities that place students at the centre of the community with assessment practices that are clear to all so that students can fully understand and evaluate their own progress” (p. 48). There is a stress on student involvement in assessing their own learning. The study points at various phases in the development of a school’s AfL culture. Teachers must be well acquainted with the goals and criteria for assessing the goals before they are ready to involve the students. This might mean that it is the teachers’ perception of the goals that are presented to the students, and less the students’ own understanding of the goals. Interestingly, when teachers feel more confident about the curriculum, the stated goals and the criteria for assessing these seem to become less important (Sandvik, 2019). The more confident teachers are, the more they seem to involve students in their own learning and assessment. The main message is that in Norway assessment is closely linked to the curriculum, and there is a strong emphasis on in-depth learning which requires students’ involvement in learning and assessment.

#### Recent innovations in AfL

At the end of Grade 10, students have exams in two subjects chosen by lottery, one written and one oral. The written exam is centralized, and the students are picked to sit the exam in one of the three subjects, math, Norwegian, or English. The second exam is locally administered and is oral and can include any of the other school subjects. Both teachers’ grade and the exam grade are written on the certificate. All students get an end of obligatory schooling certificate (end of 10^th^ grade), even if they fail in a subject.

At the end of upper secondary school, the exam system is similar. Both teachers’ grades and the exam grades are written on the certificate. In the final year, all students complete written exams in their main language which might be one of the two Norwegian languages or in Sami. In addition, all students must take three other exams, which are determined by lottery. Interestingly, even though the teacher’s grade count for 80%, the exam grade overrules the teacher’s grade. If the teacher’s grade is pass, but the exam grade is fail, the student fails the course and does not get the matric certificate. So, the teacher’s grade, which is supposed to reflect AfL has the major weight, yet does not have the decisive power.

During the pandemic all exams were canceled, and teachers’ grades formed the summative assessment, which also meant acceptance to higher education. Post pandemic, the summative exams were reintroduced, yet not without quite a lot of discussions. A large study (Sandvik et al., 2023) involving teachers and students in two major Norwegian cities found that students were not involved in the assessment process, and they felt unsupported and insecure about how their learning was assessed. Moreover, little teacher collaboration took place, and teachers struggled alone developing assessment routines without preparing for an upcoming exam. Sandvik et al.’s. (2023) study raises questions about teachers’ AfL competence which challenges equity issues when final exams are canceled. Moreover, in a smaller qualitative study (Gamlem et al., 2023) involving representatives for students, teachers, local authorities, and tertiary education leaders, the findings suggest that teachers are mainly concerned with those who missed the final exams, whereas students felt they had more time to learn instead of preparing for the exams. This was supported by tertiary education leaders who wanted more knowledgeable students. However, the issue of equity was also in this study found to be a concern.

As regards AI’s role in education, much is written in social media, it is an important theme for numerous conferences and podcasts. However, how to use AI in education and more specifically, how it affects assessment, is still an open question. The Norwegian Directorate for Education and Training (2024) have recently published a competence package for how to handle AI addressing teachers, school leaders, and local authorities. Overall, while there advantages and challenges with AI, at present, it is not sufficiently developed to act as a guideline for practitioners. The use of AI in Norwegian education is still in an early developmental stage.

#### Synergy (or lack thereof) between AfL and summative forms of assessment

A major challenge in Norway is that alongside the strong focus on AfL and teacher assessment there are national exams in reading, math and English in Grades 5, 8, and 9, in addition to the final exams in secondary school and international tests, for example, PISA. The purpose of the national exams is to provide the school with reliable information about the students’ competences in the tested subjects, and to serve AfL activities and quality development at all levels (Norwegian Directorate for Education and Training, n.d). In reality, the backwash effect of large-scale testing is stronger than the original governmental intension. There is test preparation before the tests, even in elementary school which is a gradeless system. The guidelines indicate that it might be wise to go through examples of exercises given in the tests (Norwegian Directorate for Education and Training, 2023). The latter also applies to international tests. For the final exams in secondary schools, there is a heavy focus on teaching to the test even though it makes up only 20% of the final grade. Teachers experience the tension between the strong AfL focus and the large-scale tests. The main message is that there is an intended synergy between AfL and large-scale testing in Norway; however, the reality for the teachers and students is that tension is strong and that two masters have to be served simultaneously.

### Israel

#### A decade of upheavals in education

The Israeli education system faced unprecedented instability in the past decade. Five election cycles resulted in the rise and fall of five governments and the tenure of six ministers of education, who each tried to implement a different agenda than their predecessors. Although two of the agendas were in line with the principles of AfL and had the potential to promote learning, they did not reach the implementation stage. One focused on meaningful learning (Israeli Ministry of Education, 2014) as conceived by a minister of education—a well-known educator who was dismissed before his plan was implemented. The aforementioned minister was replaced by a high-tech entrepreneur, whose disappointment with Israeli students’ attainments in international math tests drove his initiative to increase the number of students in accelerated math tracks—with the overall objective of doubling within 4 years the number of students taking five math units in the matriculation exams. Again, before reaching his goal, he was replaced by a minister of education with a different vision.

The second AfL potentially supporting agenda denotes the Matriculation Reform (Israeli Ministry of Education, 2023) in the Heritage, Social, and Humanities subjects (including literature, history, the Bible, and citizenship in the main education stream), which the elected education minister sought to advance. According to her reform, the external matriculation exams in those subjects were to be replaced by a multidisciplinary research project to be externally assessed and school-internal assessments per subject. But again, before her reform was implemented, the government changed, and the new education minister canceled it in favor of returning to traditional learning and assessment practices.

And so, the education pendulum moved from one pole to another at a dizzying speed during the political upheaval of the past decade. Hence, meaningful learning anchored in AfL did not occur nationally. Amidst this turmoil, only a small portion of schools exhibited resilience and agency thanks to their strength as school-based learning communities and their attention to professional accountability.

#### Recent trends in teaching and learning resulting from reality pressures

##### The COVID-19 pandemic and the iron sword war

Despite claims that Israel missed an opportunity to improve education following the COVID-19 pandemic, similar to warnings issued in the US against missing another chance to abolish external accountability (Braun and Marion, 2022), some lessons were learned from poor distance learning experiences during that period, which can be utilized to improve future education: Hybrid learning was implemented instead of solely relying on distance learning; the importance of involving parents in students’ learning was realized, and the need to listen to students' attitudes and consider their opinions was underscored. Furthermore, significantly increased attention has been given to social-emotional learning (SEL); expanded research and school infrastructures have been designed to implement SEL to improve education and students’ wellbeing (Benbenishty and Friedman, 2020; SEL-IL). Emphasis was also placed on helping teachers cope with the stress and anxiety caused by online teaching, and school principals were assessed in supporting their staff. Guides, tools, and professional materials for school leadership in challenging times were developed and disseminated (Avney-Rosha, n.d). All these changes formed the basis for an expansive supporting system offered to the unprecedented number of Iron Sword War victims since it broke unpredictably last October (2024). Since then, formal and informal psychological, pedagogical, and administrative guidance has been provided to schools, communities, and families to help them cope with the challenges of the harsh period and develop resilience and agency to return to normal life when the war ends.

##### Generative AI: The breakthrough of technological developments

The past year’s stormy intrusion into our lives of large language models (LLMs) that transformed AI from analytic to generative tools presents a significant challenge to the education system. These unconscious language models, which were trained on information accessible on the internet and built on the detection and statistical matching of a considerable amount of text patterns, enable authentic discourse at a linguistic level that matches that of the users and perform tasks, according to their request, at a swift speed. Therefore, the temptation is great to use these tools without engaging in critical thinking and unquestioningly accept the product produced by the model. However, the execution of the models is known to be sometimes unreliable; “hallucinations” cause them to falsify data to meet the goal and fulfill the users’ requests. In a recent article, SWATing the usage of generative AI tools for AfL, ways to maximize these models’ strengths and use them mindfully based on assignments requiring critical thinking were discussed (Birenbaum, 2023).

#### Toward an optimistic Policy Future

##### The GFN (pedagogical and managerial flexibility) reform in the education system

The new reform offers school principals and local authorities flexible budgets, managed independently, to choose from a large reservoir of external education programs those they consider necessary to cater to their students’ needs. Most expenses are covered by the State budget and a small fraction by the local authorities. Hence, the GFN reform enables principals and local authorities to exercise pedagogical and managerial responsibility to benefit their students' learning and welfare (Israeli Knesset Research and Information Center, 2023).

##### Updating RAMA’s large-scale measurement and policy

The second largest social research body in Israel, the National Assessment Authority (RAMA), established two decades ago, has performed large-scale assessment and evaluation projects to inform schools and educational policymakers since its inception. Following the Supreme Court’s ruling in 2012 in favor of disclosing achievement scores per school, the impact of the national testing program started deteriorating, and protests built up rapidly. Recently, RAMA’s CEO declared that following deep examination and lengthy debates, RAMA has shifted its focus from presenting measurement results to assessing the effectiveness of the results’ utilization for improving the clients’ decision-making as indicators of its success, hence shifting RAMA’s role from critical supervision to partnership in supporting students’ learning (Alon, 2023).

## Conclusion

It is clear from the seven country profiles of AfL that much has changed in the landscape of assessment since our seminal publication of this article in 2015 (Birenbaum et al., 2015). It is also clear that assessment does not operate in a vacuum or as an isolated policy area with the education arena. Perhaps more so than any other aspect of education, assessment in schools has been dramatically shaped by political swings, the pandemic, artificial intelligence advances, war, student and teacher diversity, and teacher professional learning and education system priorities. AfL is also challenged by the eclipsing effect of summative and large-scale assessments, which continue to occupy dominant positions in education, a position which is intensifying in some systems. Given this context, it is unsurprising that while AfL has gained some traction and has increased in prominence and prevalence across each of the seven countries, it has also struggled to become the primary method of instruction or assessment, and in some contexts has been thwarted despite its promise as a powerful pedagogy yielding significant achievement gains (see De Vries et al., 2022; William et al., 2010). Therefore, we conclude by asking, what is the future sustainability of AfL within these seven countries and beyond?

A trifecta of factors—the effects of COVID-19; the impact of AI on teaching, learning, and assessment; and the imperative for culturally responsive and personalized teaching and learning—suggests that AfL can see a renewed and optimistic future in schools. However, scholars, policymakers, and educators must push beyond AfL as a set of assessment-based practice to recognize the core values of AfL as propelling equitable, participatory communities of learning that enable personalized instruction and learning.

Lessons from COVID-19 have shown us the necessity to care for students’ wellbeing and the value of recognizing the whole child in learning and achievement. Culturally responsive pedagogy calls educators to build on and sustain students’ cultural identities through curriculum, teaching, and assessment. Doing so means honor students as individuals. Artificial intelligence compels new forms of summative assessment, ones that call for students to engage uniquely human qualities of learning—collaboration, creativity, reflection, and connection (Volante, DeLuca and Klinger, 2023a). AfL, when adopted as a powerful pedagogy, one that becomes the primary method of teaching rather than viewed as an additional assessment strategy, has the power to support and propel each of these essential goals forward.

AfL is poised to continue a productive and positive contribution to education systems and to the learning of our students. One must query whether collaborative approaches to AfL professional learning and implementation will be realized in the next decade and whether AfL will be able to meaningfully support equitable and personalized learning in the face of ongoing technological changes in a post-pandemic world.

## Notes

1. The video’s presenter on the ESA site states that AfL “is used at the beginning and during a period of instruction as teachers check for understanding.” AfL provides feedback to students and teachers to see their learning progress and where they are struggling to inform what students need to progress and what teachers need to do to enhance learning (Unpacking assessment (digitaltechnologieshub.edu.au)).

2. Writing results are returned separately later in the school year and are scored by teachers.
